# Transition Characteristic Analysis of Traffic Evolution Process for Urban Traffic Network

**DOI:** 10.1155/2014/603274

**Published:** 2014-03-27

**Authors:** Longfei Wang, Hong Chen, Yang Li

**Affiliations:** School of Highway, Chang'an University, Xi'an 710064, China

## Abstract

The characterization of the dynamics of traffic states remains fundamental to seeking for the solutions of diverse traffic problems. To gain more insights into traffic dynamics in the temporal domain, this paper explored temporal characteristics and distinct regularity in the traffic evolution process of urban traffic network. We defined traffic state pattern through clustering multidimensional traffic time series using self-organizing maps and construct a pattern transition network model that is appropriate for representing and analyzing the evolution progress. The methodology is illustrated by an application to data flow rate of multiple road sections from Network of Shenzhen's Nanshan District, China. Analysis and numerical results demonstrated that the methodology permits extracting many useful traffic transition characteristics including stability, preference, activity, and attractiveness. In addition, more information about the relationships between these characteristics was extracted, which should be helpful in understanding the complex behavior of the temporal evolution features of traffic patterns.

## 1. Introduction

Traffic congestion is increasingly becoming a serious problem for densely populated cities throughout the world. Understanding the dynamics of traffic states remains fundamental to solving diverse traffic problems. A traffic state refers to an overall description of the working state of a transportation system or its subsystems at a certain time. The present work on analysis for urban traffic state pays a considerable amount of attention to evolution analysis within region urban network [1–3]. Many researchers want to understand the dynamics of traffic transitions during the traffic evolution process in the traffic network and the conditions in which various traffic transitions emerge.

A common understanding of the fluctuations and transitions of traffic flow between different traffic regimes depends on many factors, including traffic demand, capacity of roads to carry vehicles, and the topology or structure of the network [4]. Recent studies that use differing analytical approaches have found useful characteristics and patterns in dynamical traffic evolution on urban traffic network. In these studies, the time series is widely introduced to represent the traffic state in a region by a multidimensional traffic flow vector *F*(*t*). Evaluation of the traffic network state requires analysis of how the time series of *F*(*t*) evolves with time. Reference [5] uses Kohonen's self-organizing maps (SOMs) [6] for multidimensional time series analysis. The SOM serves as a clustering algorithm as well as a dimensionality-reduction technique. Analysis of real-world traffic data shows that the method can capture the nonlinear information of traffic flows data and predict traffic flows on multiple links simultaneously. Another method [7], under the assumption that various time series representing daily cycles of traffic may be nonlinear, uses smooth-transition regression (STR) models to characterize distinct regimes for free flow, congestion, and asymmetric behavior in the transition phases from free flow to congestion and vice versa. Application tests on nonlinearity provided ample evidence of regime-dependent dynamics for all traffic time series examined. Reference [8] proposes an image-based method of observing and analyzing traffic state over large road network. Urban traffic network is mapped to a pseudocolor image to vividly represent the macroscopic traffic state. The evolutionary patterns of traffic state are determined by calculating and analyzing the optical flow field of consecutive pseudocolor images; thus, the congested regions can be found automatically.

Recent studies on traffic state analysis, including validation of traffic flow models [9], investigation of traffic patterns [10], recognition of traffic congestion propagation [11], and classification of urban traffic state [12], demonstrate that dynamical properties of traffic flow on urban traffic networks have some traffic patterns during different periods of a day. The traffic pattern, generally defined as a set of traffic states with characteristics of traffic flow, average vehicle speed, and occupancy, represents a particular state with the same special and temporal characteristics [13]. Many authors emphasize the importance of finding models by partitioning the traffic states into patterns and using fuzzy reasoning [14], clustering analysis [15, 16], dimensionality reduction [17], and data fusion [18] to investigate their properties. However, previous studies on pattern-oriented traffic state analysis lack attention to the transition characteristic of traffic state pattern during traffic evolution process.

Because of the shortage of studies that consider the transition characteristic between traffic patterns in the evolution process on urban traffic network, the main objective of this study is to investigate the temporal characteristics and distinct regularity in the traffic evolution process from the viewpoint of the whole network. Our particular interest is to understand when and how the traffic state transitions occur on traffic networks with the fluctuations of traffic flow.

In this paper, we construct a transition network model of traffic state pattern and have attempted to gain in-depth insights into the evolution characteristic of traffic time series. Furthermore, a close look at transition characteristic of traffic state pattern not only provides useful information for application in ITS, such as incident detection, extended delay prevention, and traffic control scheme, but also improves upon the shortage of conventional models that lack measurement and quantization of traffic evolution. Besides what was previously stated, we believe that this analysis provides direction for new steps in understanding the complex behavior of the temporal evolution features of traffic patterns. To illustrate the methodology, we use flow rate data of multiple road sections from Network of Shenzhen's Nanshan District, China.

The following content of the paper begins with the rationale for methodology, followed by a brief on data acquisition and a discussion of analytical results. We conclude with an elaboration on the implications of our research and suggestions for a future agenda.

## 2. Traffic State Network Analysis Model

In order to conduct quantitative analysis of the traffic evolution process, a base network model for traffic state analysis of urban regional network is constructed to describe the transition relationship of traffic states. Here we introduce the following notions.

**Definition 1**

The regional traffic state during time interval *t* can be represented by a *d*-dimensional vector, as shown in

(1) $$\begin{array}{c} F\left(t\right)={\left[f_{1}\left(t\right),f_{2}\left(t\right),\ldots ,f_{n}\left(t\right)\right]}^{T}, \end{array}$$

where *f*_*i*_(*t*) represents the flow rate of the *i*th section within the time period *t* and *n* represents the total number of all sections. *F*(*t*) represents the traffic state in this time interval. As the traffic state changes with time *t*, the flow rates on the different links vary as reflected in the multidimensional time series *F*(*t*).

Similar traffic states could repeat occurrences at different times. This leads to the idea of performing clustering on the traffic state to identify large clusters as traffic patterns.

**Definition 2**

According to cluster analysis of the total gathered traffic state data set, multiple traffic state classifications are obtained. Each classification is a set of traffic states, which is defined as traffic state pattern.

**Definition 3**

If *P*_A_ and *P*_B_ are traffic state patterns, *F*(*t*_1_) ∈ *P*_A_,  *F*(*t*_2_) ∈ *P*_B_,  and  *t*_1_ and *t*_2_ are adjoining times, then there is a traffic evolution temporal relation between *F*(*t*_1_) and *F*(*t*_2_). In addition, there is traffic state pattern transition relation (TSPTR) between *P*_A_ and *P*_B_, denoted by *R*_A→B_ = *P*_A_ → *P*_B_.

**Definition 4**

The directed network, with traffic state pattern as vertex and traffic state pattern transition relation as edge, is called traffic state pattern transition relation network (TSPTRN), denoted by *G* = (*P*, *R*), where *P* represents all traffic state pattern sets, and *R* represents all transition relation sets.

**Figure 1:**
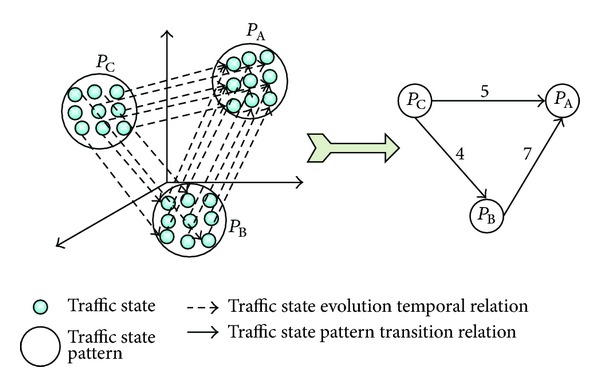
The structure diagram of TSPTRN.

TSPTRN can be constructed by traffic state data accumulated within a period of time. The weight *w*_A→B_ of *R*_A→B_ = *P*_A_ → *P*_B_ is determined by the total number of evolution temporal relations between all traffic states of *P*_A_ and *P*_B_.  Figure 1 shows the structure of TSPTRN, with transition relations of three traffic state patterns, namely, *P*_A_, *P*_B_, and *P*_C_.

## 3. Analysis of the Features of Traffic State Pattern Transition

In this section, we use the TSPTRN model to analyze the traffic transition characteristics of state pattern transition in the traffic evolution process, including stability, preference, activity, and attractiveness.

### 3.1. The Stability of Traffic State Pattern

The stability of traffic state pattern consists of the following two parts:

1. *balance ability*: the ability to maintain one state,
2. *recovery ability*: the ability to transit back to the original pattern in the next period after having transited from one pattern to another.

**Definition 5**

From time period 1 to *N*, traffic operation experiences traffic state sequence *L* = *S*_1_, *S*_2_,…*S*_*N*_. If all of these *N* states belong to traffic state pattern *P*, that is, pattern *P* remains the same for *N* consecutive time periods, then *L* is called the consecutive state sequence of *P*, denoted by *L*_*P*_; set *N* as the consecutive coefficient of *L*_*P*_, denoted by C_*L*_*P*__.

**Definition 6**

Set *L* = *S*_1_, *S*_2_,…*S*_*N*_ as the consecutive state sequence of traffic state pattern *P*, if it meets all of the following conditions:

1. *L* has states that belong to *P* and also has states that do not belong to *P*;
2. *S*_1_ and *S*_*N*_ belong to *P*;
3. states in *L* that do not belong to *P* are not consecutive.

Then *L* is called the approximate consecutive state sequence of *P*, denoted by ${\tilde{L}}_{P}$. In ${\tilde{L}}_{P}$, the number of states that do not belong to *P* is denoted by *M*, and set the consecutive coefficient of ${\tilde{L}}_{P}$ as ${\text{C}}_{{\tilde{L}}_{P}}$, as shown in

(2) $$\begin{array}{c} {\text{C}}_{{\tilde{L}}_{P}}=N\times {\left(1-\omega \right)}^{M}. \end{array}$$

The calculation method for the stability of traffic state pattern *P* is as follows: first, isolate all consecutive state sequences and approximate consecutive state sequences of *P* in the total data set; second, denote the arithmetic mean of consecutive coefficients of all these sequences by the stability coefficient *γ*_*S*_^*P*^ of *P*, as shown in formula (3), where *N* and *M* are the total number of all consecutive state sequences and all approximate consecutive state sequences, respectively. With greater *γ*_*S*_^*P*^ value, *P* is more stable. Consider

(3) $$\begin{array}{c} \gamma_{S}^{P}=\frac{\sum_{i=1}^{N}{\text{C}}_{L_{P}}^{i}+\sum_{j=1}^{M}{\text{C}}_{{\tilde{L}}_{P}}^{j}}{N+M}. \end{array}$$

### 3.2. Preference of Traffic State Pattern

Preference refers to the tendency of traffic state pattern transiting from one to another. For traffic state pattern *P*_A_, if the transition time among traffic states within *P*_A_ is ${\hat{n}}_{P}$ and transition time among traffic states between *P*_A_ and *P*_B_, *P*_C_, and *P*_D_ is *n*_A→B_, *n*_A→C_, and *n*_A→D_, respectively, then the probability of *P*_A_ remaining unchanged in the next period is shown in

(4) $$\begin{array}{c} {\text{TP}}_{P_{\text{A}}}^{\mathrm{In}}=\frac{{\hat{n}}_{P}}{{\hat{n}}_{P}+n_{\text{A}\to \text{B}}+n_{\text{A}\to \text{C}}+n_{\text{A}\to \text{D}}}\times 100\%. \end{array}$$

The probability for *P*_A_ transiting to other traffic state patterns in the next period is shown in

(5) $$\begin{array}{c} {\text{TP}}_{P_{\text{A}}}^{\text{Out}}=\frac{n_{\text{A}\to \text{B}}+n_{\text{A}\to \text{C}}+n_{\text{A}\to \text{D}}}{{\hat{n}}_{P}+n_{\text{A}\to \text{B}}+n_{\text{A}\to \text{C}}+n_{\text{A}\to \text{D}}}\times 100\%. \end{array}$$

If *P*_A_ transits to another pattern in the next period, the probabilities of transiting to *P*_B_,  *P*_C_, and *P*_D_ (i.e., the transition preference of *P*_A_ to *P*_B_,  *P*_C_, and *P*_D_), taking *P*_B_ as an example, are shown in

(6) $$\begin{array}{c} {\text{TP}}_{P_{\text{A}}\to P_{\text{B}}\;|\;\text{Out}}^{\text{Out}}=\frac{n_{\text{A}\to \text{B}}}{n_{\text{A}\to \text{B}}+n_{\text{A}\to \text{C}}+n_{\text{A}\to \text{D}}}\times 100\%. \end{array}$$

According to a conditional probability formula, the probability of *P*_A_ transiting to *P*_B_ in the next period is shown in

(7) $$\begin{array}{c} {\text{TP}}_{P\to \text{A}}^{\text{Out}}={\text{TP}}_{P_{\text{A}}\to P_{\text{B}}\;|\;\text{Out}}^{\text{Out}}\times {\text{TP}}_{P_{\text{A}}}^{\text{Out}}. \end{array}$$

### 3.3. The Activity of Traffic State Pattern

The out-degree of *P*_A_ represents its activity. If the out-degree is higher, then *P*_A_ is easier to transit to other state patterns with higher activity.

Define the external transition times of traffic state pattern *P*_*i*_ as TO_*P*_*i*__ = ∑_*j*=1_^*M*_*i*_^ *ω*_*P*_*i*__^*j*^, where *ω*_*P*_*i*__^*j*^ is the weight of each outgoing edge *P*_*i*_, and *M*_*i*_ is the out-degree of *P*_*i*_. Rank the times of external transition of all traffic state patterns in an ascending order, and then the distribution probability *P*_TO_*P*_*i*___ for each of external transition times can be obtained, as in

(8) $$\begin{array}{c} P_{{\text{TO}}_{P_{i}}}=\frac{{\text{TO}}_{P_{i}}\times n_{P_{i}}}{\sum_{i=1}^{N}{\text{TO}}_{P_{i}}\times n_{P_{i}}}, \end{array}$$

where *N* is the total number of different external transition times and *n*_*P*_*i*__ is the number of all traffic state patterns with the same external transition time as TO_*P*_*i*__; thus, the expected value of each external transition time could be obtained, as in

(9) $$\begin{array}{c} E\left({\text{TO}}_{P_{i}}\right)=\sum_{i=1}^{N}\;{\text{TO}}_{P_{i}}\times P_{{\text{TO}}_{P_{i}}}. \end{array}$$

Define the activity coefficient of *P*_*i*_ as *γ*_A_^*P*_*i*_^, as in formula (10). The greater is the coefficient value, the stronger is the activity. Consider

(10) $$\begin{array}{c} \gamma_{\text{A}}^{P_{i}}=e^{\left({\text{TO}}_{P_{i}}-E({\text{TO}}_{P_{i}})\right)/\mathrm{Max}({\text{TO}}_{P_{i}})}. \end{array}$$

### 3.4. The Attractiveness of Traffic State Pattern

The in-degree of *P*_A_ represents the attractiveness of *P*_A_. If the in-degree of *P*_A_ is higher, then other state patterns are easier to transit to *P*_A_ with attractiveness.

Define the internal transition times of traffic state pattern *P*_*i*_ as TI_*P*_*i*__ = ∑_*j*=1_^*M*_*i*_^ *ω*_*P*_*i*__^*j*^, where *ω*_*P*_*i*__^*j*^ is the weight of each incoming edge of *P*_*i*_, and *M*_*i*_ is the in-degree of *P*_*i*_. Rank the internal transition times of all traffic state patterns in an ascending order, and then the distribution probability *P*_TI_*P*_*i*___ of each internal transition times could be obtained, as in

(11) $$\begin{array}{c} P_{{\text{TI}}_{P_{i}}}=\frac{{\text{TI}}_{P_{i}}\times n_{P_{i}}}{\sum_{i=1}^{N}{\text{TI}}_{P_{i}}\times n_{P_{i}}}, \end{array}$$

where *N* is the total number of different internal transition times and *n*_*P*_*i*__ is the number of all traffic state patterns with the same internal transition times as TO_*P*_*i*__; thus, the expected value of each internal transition times could be obtained, as

(12) $$\begin{array}{c} E\left({\text{TI}}_{P_{i}}\right)=\sum_{i=1}^{N}\;{\text{TI}}_{P_{i}}\times P_{{\text{TI}}_{P_{i}}}. \end{array}$$

Define the attractiveness coefficient of *P*_*i*_ as *γ*_B_^*P*_*i*_^, as in formula (13). The greater is coefficient value, the bigger is the attractiveness of the activity. Consider

(13) $$\begin{array}{c} \gamma_{\text{B}}^{P_{i}}=e^{\left({\text{TI}}_{P_{i}}-E({\text{TI}}_{P_{i}})\right)/\mathrm{Max}({\text{TI}}_{P_{i}})}. \end{array}$$

## 4. Experiment

**Figure 2:**
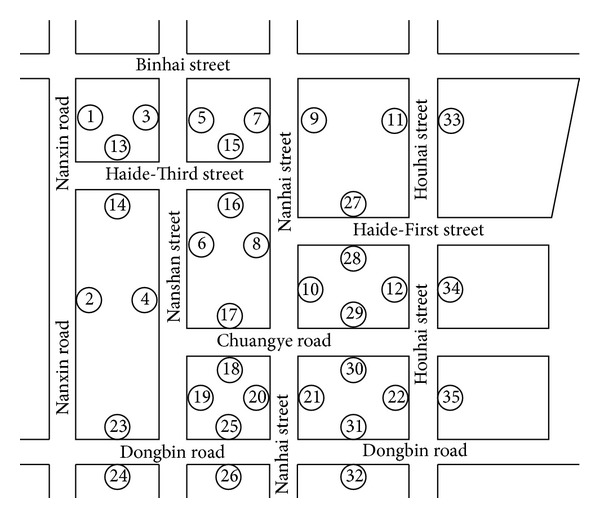
Schematic diagram of Nanshan road network topology.

This paper examines the road network of Shenzhen's Nanshan District to record the flow rates (vehicles per hour (veh/h)) of each road segment every 15 min. The road network map is sketched in Figure 2. The network flow rates of 35 road segments (*d* = 35) were measured from June 1st to June 30th, 2011. Thus, the experimental dataset consists of flow rates for 6,480 consecutive time intervals on 35 links, defining a 35-dimensional series with a length of 6,480.

### 4.1. Analysis of Traffic State Pattern Transition

**Figure 3:**
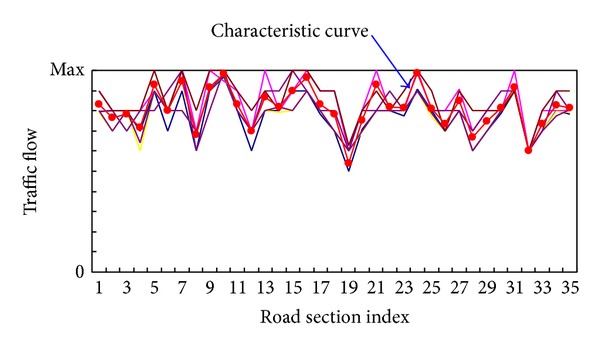
The characteristic curve of traffic state mode.

This experiment uses Kohonen's self-organizing maps (SOMs) [5] for the multidimensional time series analysis. A well-trained 8∗8 SOM network is used to cluster all the data. The SOM here serves as a clustering algorithm as well as a dimensionality-reduction technique when applied to cluster the traffic flow vectors. After the SOM was trained, 64 clusters were obtained. Each cluster represents a traffic state pattern with 35 dimensions. Through fitting all the traffic state curves within each pattern using mean fitting method, we get the characteristic curve of each traffic state pattern, as shown in Figure 3.

**Figure 4:**
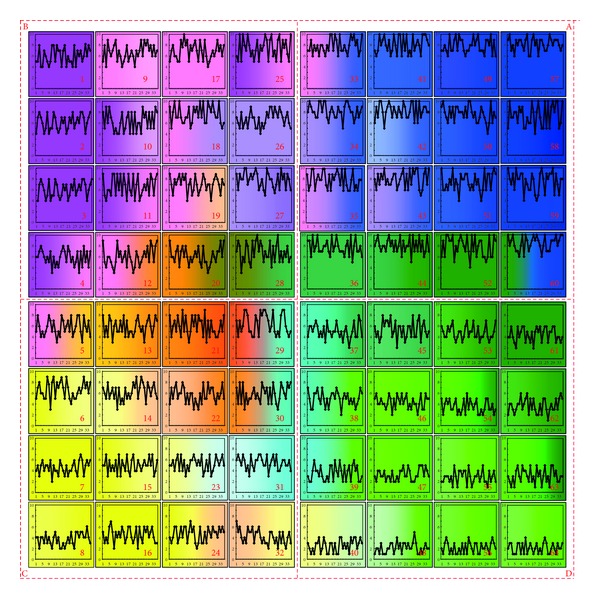
Schematic diagram of 64 traffic state patterns.

The distribution diagram of all traffic state patterns is shown in the SOM topological grid in Figure 4 and each pattern is dyed by a special color and has a unique characteristic curve. It could be observed that adjacent clusters represent similar patterns and each pattern changes gradually along the SOM grid. Four pattern groups A, B, C, and D are shown in this figure, respectively. For pattern group A of “blue clusters,” all links have very large flow rates and even traffic distribution. For pattern group B of “pink clusters,” the flow rates are a little lower and less evenly distributed among the links, compared to the “blue clusters” of area A. For pattern group C of “yellow clusters,” the links have moderate flow rates and uneven traffic distribution, with several links in some clusters having high flow rates in spite of the low flow rates in other links. For pattern group D of “green clusters,” the links have small flow rates and even traffic distribution.

**Figure 5:**
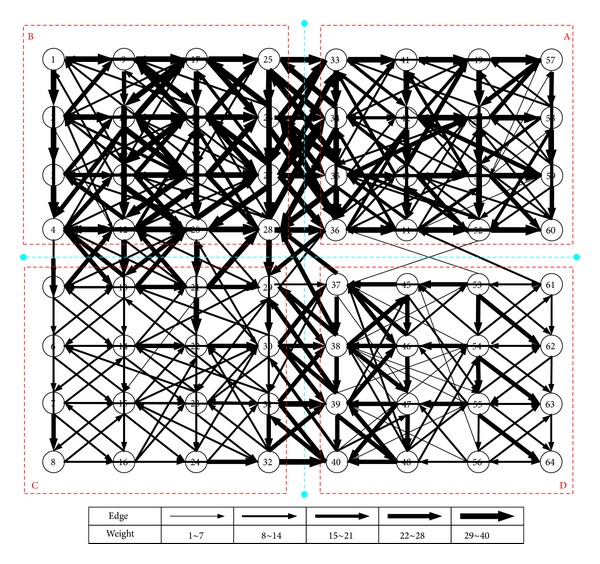
Traffic state pattern transition relation network (TSPTRN) of Shenzhen's Nanshan District.

The traffic state pattern transition relation network (TSPTRN) of Shenzhen's Nanshan District is drawn in Figure 5. We can clearly see all the transition relations of total 64 patterns. By statistical analysis to TSPRN, all traffic state patterns, transition times, major time, and traffic features of four pattern groups A, B, C, and D are obtained, respectively, as shown in Table 1.

According to Table 1, the distribution time of A, B, C, and D pattern groups is relatively fixed, indicating that macroscopic traffic operation of the road network has strong regularity and the traffic operation is stable within a certain period of time. Because the distribution time directly corresponds to the transition time of traffic state, traffic state transition times of groups A and B are significantly larger than those of groups C and D.

According to Table 1 and Figure 5, pattern transition is mainly distributed in groups A and B. This is because traffic patterns of groups A and B account for 77.8% of that in the whole day, and thus the pattern transition time is relatively larger while that of groups C and D is mainly in the early morning and at night, and thus the pattern transition time is relatively smaller.

However, for the proportion of pattern transition among pattern groups, the proportions of groups B and C are slightly greater than those of groups A and D. This is because groups A and D are, respectively, at peak hours and free travel period with relatively stable traffic demand and traffic distribution without significant disturbance.

In addition, the connection between pattern groups is also an area with frequent transition, accounting for 19.23% of the total pattern transition, and major transition occurs between groups A and B, accounting for 89.2% of the total critical transition.

### 4.2. Analysis of the Characteristics of TSPTRN

**Figure 6:**
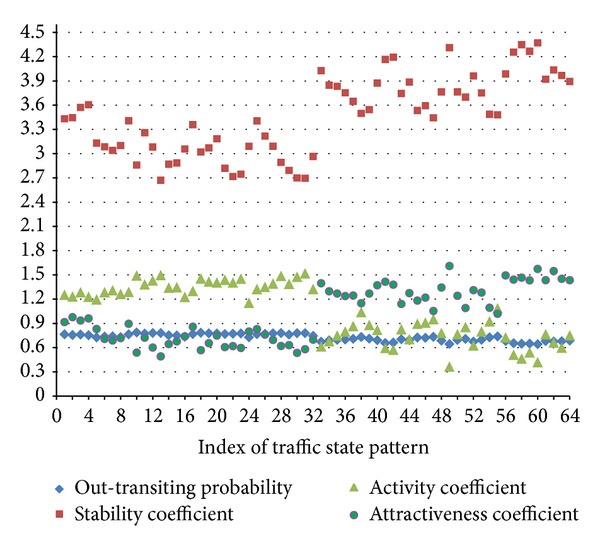
Characteristic values distribution of traffic state pattern transition.

**Figure 7:**
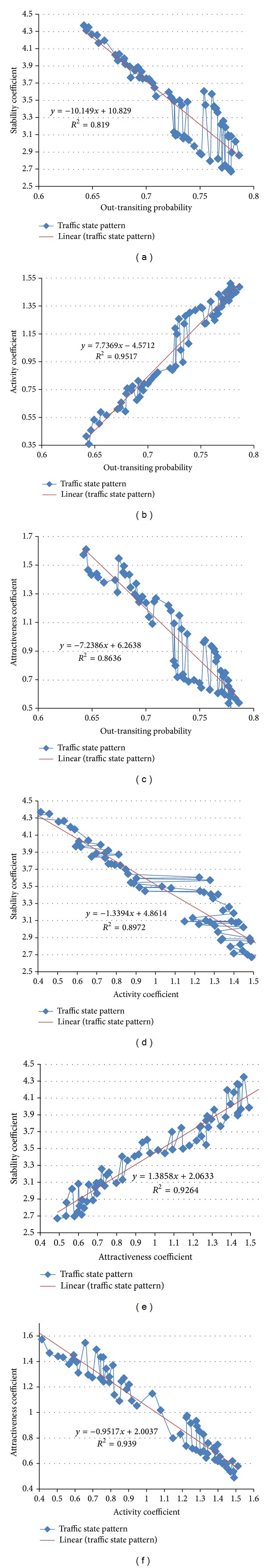
The relationships between traffic transition characteristics coefficients.

The detailed characteristics data of each traffic state pattern within TSPTRN are shown in Figure 6. The relations between 4 traffic transition characteristics coefficients are, respectively, shown in Figure 7.

There are obvious linear increasing or decreasing relationships between these characteristics. We can clearly see that the stability coefficient increases with the attractiveness coefficient and that both of them decrease with the out-transiting probability and the activity coefficient. This illustrates that, in the traffic state pattern transition process, the stability and attractiveness, which reflect the static characteristic, are in opposition to out-transiting probability and activity, which reflect the dynamic characteristic.

The mean values of each traffic state pattern group within TSPTRN are shown in Table 2, respectively.

According to Table 2, the external transition probability of the traffic state pattern of group A is relatively smaller than that of others, while its stability coefficient and attractiveness coefficient are relatively larger. This is because group A is in the morning peak and evening peak hours, the traffic state of each road is in the “saturated” and “nearly saturated” condition, and the change interval of the traffic state is limited.

The traffic state pattern of group B is near the morning peak and evening peak, with a very unstable traffic operation state. Thus, it has a larger average external transition probability, poorer stability, stronger average activity, and a relatively smaller attractiveness coefficient.

The traffic state patterns of group C are before and after the patterns of group B. During these periods, the traffic operation state is at a transition stage from instability to stability with significant changes. So the traffic operation state has a relatively larger average external transition probability and activity coefficient and a smaller stability and attractiveness coefficient.

The traffic state pattern of group D happens in the early morning and late at night. In these periods, the traffic is basically at the state of free travel, and the traffic operation state is quite smooth and stable. Thus, it has a larger stability coefficient and attractiveness coefficient and a relatively smaller average external transition probability and activity coefficient.

## 5. Conclusions

In this paper, we have investigated the transition characteristics and distinct regularity of traffic state pattern during the traffic evolution process from the viewpoint of the whole network. A transition network model of traffic state pattern is constructed, which could facilitate gaining in-depth insights into the evolution characteristic of traffic time series. According to our empirical results, the proposed analytical method permits extracting more information on traffic transition characteristics in the constructed traffic state pattern spaces, particularly including stability, preference, activity, and attractiveness. These favorable features of our method make it a potentially powerful tool for traffic evolution analyses of urban regional networks.

However, in contrast with the temporal transition characteristics of traffic state patterns that occur only in time, spatiotemporal transition characteristics that occur in both time and space can also be investigated. Methodologies taking topology structure and traffic demand into account may gain usefulness, so we intend to further investigate other spatiotemporal transition characteristics in future research.

## Figures and Tables

**Table 1:** A detailed table of the transition characteristics of pattern groups.

Pattern group	Traffic state transition times	Pattern transition times	Pattern transition ratio	Occurrence time	Time distribution
A	2325	1528	65.7%	7:30~11:00 16:00~19:30	38.89%
B	2329	1740	74.7%	7:00~7:30 11:00~16:00 19:30~21:00	38.89%
C	850	649	76.3%	6:30~7:00 21:00~22:00	8.33%
D	976	670	68.6%	6:00~6:30 22:00~24:00	13.89%

Total average	6480	4588	70.8%		

**Table 2:** Table of detailed characteristics of pattern group transition.

Pattern group	Average external transition probability	Average stability coefficient	Average activity coefficient	Average attractiveness coefficient
A	0.6737	4.02	0.6243	1.3476
B	0.7703	3.24	1.3518	0.7668
C	0.7526	2.89	1.3452	0.6592
D	0.7039	3.71	0.8347	1.2858

Total average	0.7302	3.46	1.042	1.017
